# Navigating the digital frontier during the COVID‐19 pandemic and EVALI epidemic: The impact of social media on public health

**DOI:** 10.1002/puh2.173

**Published:** 2024-04-17

**Authors:** Chimwemwe Ngoma, Apatsa Matatiyo, Gabriel Ilerioluwa Oke, Yusuff Adebayo Adebisi, Deborah Oluwaseun Shomuyiwa

**Affiliations:** ^1^ Tobacco Harm Reduction Scholarship Programme, Knowledge•Action•Change London UK; ^2^ ThinkSmart Consulting Lilongwe Malawi; ^3^ Paediatric Cancer Program University of North Carolina Project ‐ Malawi Lilongwe Malawi; ^4^ Department of Healthcare Management University of Global Health Equity Kigali Rwanda; ^5^ Research Department Global Health Focus Africa Abuja Nigeria; ^6^ Faculty of Pharmacy University of Lagos Lagos Nigeria

**Keywords:** COVID‐19, EVALI, health crises, pandemic, public health, social media

## Abstract

This commentary explores how social media influenced public health narratives and responses during two recent health crises, the COVID‐19 global pandemic and the e‐cigarette or vaping product use–associated lung injury (EVALI) epidemic. In the context of COVID‐19 pandemic, social media played a dual role, acting as both a catalyst for information dissemination and a breeding ground for misinformation. This situation highlighted the challenges public health institutions face in navigating online narratives and maintaining public trust during crises. Conversely, the EVALI epidemic shed light on how social media narratives can impact public perception and policy decisions. Initially, attributed to nicotine vaping, subsequent investigations revealed that contaminated cannabis oils containing vitamin E acetate were the actual cause, leading to shifts in public discourse and regulatory considerations. This case underscores the importance of accurate information dissemination to prevent misinterpretations that could negatively impact public health interventions. The commentary also highlights the evolving nature of online narratives and the need for public health agencies to adapt their strategies continually. Fact‐checking programs, public health campaigns, and collaborations with technology companies emerged as critical strategies to combat misinformation and promote evidence‐based practices. However, addressing the root causes of misinformation, such as addressing distrust in institutions, remains an ongoing challenge requiring multifaceted approaches. Looking ahead, controlling online narratives during health crises will continue to present challenges as social media platforms evolve and new challenges emerge. Finding a balance between information sharing and privacy protection while fostering digital literacy skills among the public will be paramount. Collaborative efforts among public health agencies, technology companies, and communities will be essential in navigating and managing online narratives effectively. This commentary underscores the interplay between social media and public health, highlighting the need for strategic interventions, accurate information dissemination, and ongoing adaptation to address emerging challenges in the digital age.

## BACKGROUND

The emergence of social media has brought about a transformative era in which health information is disseminated at an unprecedented pace. The public often turns to social media for information during emerging health issues and infectious disease outbreaks [1]. Over the past decade, social media has significantly contributed to disseminating knowledge and raising awareness about public health issues [2]. It has evolved into a space where discussions about disease symptoms, prognoses, medical examinations, procedures, and treatments thrive [3]. However, social media is a conundrum, facilitating both the rapid dissemination of credible information and the propagation of misinformation [4].

As the 2019 e‐cigarette or vaping product use–associated lung injury (EVALI) epidemic in the United States of America (USA) and the global COVID‐19 pandemic emerged, social media turned into a breeding ground for misleading rumors and conspiracy theories [5]. COVID‐19, caused by SARS‐CoV‐2, led to a global crisis with rapid human‐to‐human transmission. In contrast, EVALI, associated with tetrahydrocannabinol cartridges, was erroneously linked to nicotine vaping initially and later connected to contaminated cannabis oils [6].

In the case of the COVID‐19 pandemic, narratives were predominantly driven by social media and linked with fear‐mongering about the severity of the disease and panic‐instigated consumer behavior [7]. This was evident in instances of panic buying of drugs and household drug stockpiling [8]. Conversely, during the EVALI epidemic, the initial narratives primarily focused on attributing the disease to nicotine vaping, influencing public perception and policy decisions [9].

This commentary explores the influence of social media on public health narratives and responses during the COVID‐19 pandemic and EVALI epidemic. The two crises serve as compelling examples of how the digital realm has fundamentally changed the landscape of public health. Social media has not only influenced public opinion but also steered behavioral changes. This is evident in practices ranging from mask‐wearing and vaccination to the banning of e‐cigarettes.

## THE INFLUENCE OF ONLINE NARRATIVES ON PUBLIC RESPONSE

Within the digital landscape, narratives evolve and hold significant power over public comprehension of events such as in the case of COVID‐19 and EVALI. In this context, online narratives can be described as the collective posting and reposting of interactions on digital platforms involving the retelling and responding to large‐scale mediated events [10]. These narratives play an important role in shaping public perception and influencing behavior during health crises.

For instance, during the COVID‐19 pandemic, the public's desperation for information led to a surprising abundance of information and conspiracy theories about the pandemic on social media platforms [11]. A striking thing about the COVID‐19 crisis was the convergence of virology and virality. Not only did the virus itself spread rapidly, but so did the information—and misinformation—about the outbreak, fanning the flames of public panic. The spread of panic on social media often outpaced the actual spread of COVID‐19 [7]. This phenomenon is not uncommon during disease outbreaks, particularly when traditional media do not provide relevant, timely information for the public and less credible information from public health officials [12].

Other narratives downplayed the severity of the virus, fostering misconceptions about its origins and modes of transmission. Such narratives fueled public skepticism toward public health measures such as mask‐wearing and vaccination. Furthermore, some early messages from trusted experts were later proven to be inadequate or incorrect [13]. However, as the pandemic progressed, evidence‐based information gradually replaced these narratives. Official social media also became a vital critical channel for the public to access pandemic information [4], emphasizing the importance of social distancing, mask usage, and vaccination in curbing the virus's spread.

In contrast, during the EVALI epidemic, the initial attribution by the Centers for Disease Control and Prevention linked the epidemic to nicotine vaping due to its correlation with increased youth nicotine vaping in the USA. However, subsequent case–control studies revealed that the majority of EVALI cases were linked to contaminated illicit cannabis oils containing vitamin E acetate [9]. Content analysis studies found a significant uptick in tweets related to negative health outcomes of nicotine vaping during the EVALI epidemic [14].

Consequently, some smokers who had switched from smoking to e‐cigarettes as a safer alternative might have switched back to smoking out of the mistaken belief that EVALI was caused by vaping nicotine [15]. Government regulatory proposals, including the consideration of bans on the sale of nicotine flavors and vaporizers, were also founded on the assumption that nicotine vaping was the primary cause of the lung injuries [9]. This illustrates how public health authorities were forced to make definitive statements when they might not have been fully prepared, likely due to the public's heightened demand for information.

These two scenarios underscore the influence that online narratives wield over public perception and policymaking. In both the cases of EVALI and the COVID‐19 pandemic, online narratives played a role in shaping early responses. This highlights the urgent need for rapid and accurate dissemination of evidence‐based information in the digital age, as these narratives have significant power and can have a profound impact on policy decisions. Whether EVALI or COVID‐19, addressing complex public health issues depends on navigating and managing online narratives within the digital sphere.

## NAVIGATING ONLINE NARRATIVES DURING HEALTH CRISES

Throughout both crises, public health agencies faced an array of challenges when managing information. These challenges include a lack of control over the narrative, the rapid dissemination of misinformation, and a declining trust in official sources among the public. These challenges emerged as pressing concerns due to the evolving nature of the crises and the associated urgency of accurate information dissemination. In response to these challenges, public health agencies implemented multifaceted strategies that hold valuable lessons for future crises.

One key strategy involved the stepping up of fact‐checking programs to combat misinformation [16]. For example, Facebook, Twitter, and YouTube concentrated their efforts in the early stages of the pandemic on limiting the visibility of COVID‐19 misinformation on their platforms through policies and practices rife with subjectivity [17]. The initiative rigorously examined the veracity of information being shared on digital platforms, dispelling false narratives and encouraging the spread of reliable information. However, it is worth noting that addressing misinformation that has permeated public consciousness poses unique challenges. Even when fact‐checking is accurate and timely, there may be cognitive biases and social dynamics that contribute to the persistence of misinformation [18]. This underscores the need for subtle and sophisticated approaches in tackling misinformation.

Additionally, extensive public health campaigns that addressed urgent public health issues as well as distributed authoritative guidance and evidence‐based practices were carried out with the dual goals of educating the public about developing health crises and reducing the impact of pervasive misinformation. Official social media channels belonging to public health agencies also emerged as indispensable tools that served as conduits for indirectly shaping information flow, providing a steady stream of pandemic‐related updates while elevating the accuracy and quality of shared information [4]. This was achieved through the continuous provision of accurate pandemic information and the ongoing enhancement of its quality.

Recognizing the importance of collaboration, public health agencies forged partnerships with technology companies to develop algorithms prioritizing credible information. One noteworthy initiative in this regard is the Organized Search Results Panel (OSRP), jointly launched by Google and the World Health Organization (WHO) in response to the COVID‐19 pandemic. The OSRP was designed to ensure that users have access to reliable information exclusively. The platform curates information from authoritative sources, such as the WHO, national governments, health agencies, and a select list of reputable news outlets [19]. To further bolster response strategies, several countries and public health agencies harnessed the power of data analytics and monitoring tools during the COVID‐19 pandemic to offer real‐time insights into the dissemination of misinformation and public sentiment, empowering agencies to adapt their communication strategies and effectively counteract the influence of misleading information [20]. By addressing the rapid spread of misinformation, asserting control over the narrative, and rebuilding public trust, governments and health authorities can fulfill their mission of safeguarding public well‐being during health emergencies.

## FUTURE CHALLENGES

Looking ahead, controlling online narratives during health crises may present new challenges for public health authorities. Unexpected challenges may arise as a result of the social media platforms’ rapid expansion and the emergence of new platforms. Finding the ideal balance between information sharing and privacy protection will continue to be a delicate endeavor. Additionally, addressing the root causes of misinformation, such as addressing systemic issues that foster distrust in institutions, remains a complex and long‐term challenge. Governments and public health authorities must be prepared to work with diverse communities and adapt their strategies to different cultural and social contexts.

## CONCLUSION

The intertwined relationship between social media and public health crises, as exemplified by the COVID‐19 pandemic and the EVALI epidemic, underscores the urgent need for strategic interventions to mitigate the spread of misinformation while promoting evidence‐based narratives. It is paramount to integrate robust, real‐time fact‐checking mechanisms and enhance the public's digital literacy skills to discern credible information amidst the plethora of content online. Collaborative efforts among public health agencies, technology companies, and the global community are essential to develop adaptive algorithms and platforms that prioritize and amplify accurate, evidence‐based information. Moreover, fostering a culture of transparency and responsiveness in communication will be crucial in rebuilding public trust and enhancing the effectiveness of health communication in the digital age.

## AUTHOR CONTRIBUTIONS

The original concept for this manuscript was conceived by Chimwemwe Ngoma and Apatsa Matatiyo. Chimwemwe Ngoma took the lead in composing the initial draft. Subsequently, Chimwemwe Ngoma, Gabriel Ilerioluwa Oke, Deborah Oluwaseun Shomuyiwa, Yusuff Adebayo Adebisi, and Apatsa Matatiyo engaged in a critical revision process to enhance its intellectual depth. All authors participated in reviewing, reading, and endorsing the final version of the paper.

## CONFLICT OF INTEREST STATEMENT

Chimwemwe Ngoma is a consultant to Knowledge•Action•Change (K•A•C). K•A•C is a public health organization with a focus on tobacco harm reduction and is funded with a grant from the Foundation for a Smoke‐Free World, Inc. (FSFW), a US nonprofit 501(c)(3) private foundation. Gabriel Ilerioluwa Oke and Yusuff Adebayo Adebisi are scholars in the Tobacco Harm Reduction Scholarship Programme (THRSP) by K•A•C. Deborah Oluwaseun Shomuyiwa and Yusuff Adebayo Adebisi are Youth Editorial Board members of Public Health Challenges and co‐authors of this article. To minimize bias, they were excluded from all editorial decision‐making related to the acceptance of this article for publication. The remaining author declares no competing interests.

## FUNDING INFORMATION

The authors did not receive any funding to develop this paper.

## Data Availability

All sources used in this manuscript have been duly cited.
